# Time Unpacking Effect on Intertemporal Decision-Making: Does the Effect Change With Choice Valence?

**DOI:** 10.3389/fpsyg.2021.666329

**Published:** 2021-05-28

**Authors:** Quan Yang, Xianmin Gong, Jinli Xiong, Shufei Yin

**Affiliations:** ^1^Department of Psychology, Hubei University, Wuhan, China; ^2^Big Data Decision Analytics Research Centre, Department of Psychology, The Chinese University of Hong Kong, Hong Kong, China; ^3^CAS Key Laboratory of Mental Health, Institute of Psychology, Chinese Academy of Sciences, Beijing, China; ^4^Beijing Key Laboratory of Applied Experimental Psychology, Faculty of Psychology, Beijing Normal University, Beijing, China

**Keywords:** intertemporal choice, temporal discounting, time unpacking effect, emotional valence, time perception

## Abstract

People often feel that a period of time becomes longer when it is described in more detail or cut into more segments, which is known as the time unpacking effect. The current study aims to unveil how time unpacking manipulation impacts intertemporal decision making and whether the gain-loss valence of choices moderates such impacts. We recruited 87 college students (54 female) and randomly assigned them to the experimental conditions to complete a series of intertemporal choice tasks. The subjective values of the delayed choices were calculated for each participant and then analyzed. The results showed that participants perceived longer time delays and higher subjective values on the delayed gains (but not losses) in the time unpacking conditions than in the time packing conditions. These results suggest that time unpacking manipulation not only impacts time perception but also other factors, which in turn, influence the valuation of delayed outcomes and thereby intertemporal choices. The results are discussed in comparison to previous studies to highlight the complexity of the mechanism underlying the effect of time unpacking on intertemporal decision making.

## Introduction

Intertemporal decision-making refers to the process of weighing and choosing outcomes that occur at different points in time, such as a short-term vs. a long-term benefit, writing a manuscript today vs. next week (Frederick et al., 2002). As a critical aspect of intertemporal choice, time significantly influences the decisions people make. The time unpacking effect, a relatively newly identified phenomenon, shows that framing time in an unpacking manner (e.g., describing more events and remarks on the timeline or cutting a time interval into a larger number of small segments) extends the length of time interval people perceive. It is thus reasonable to speculate that the manipulation of unpacking time may influence people’s time perception and thereby their intertemporal decision-making. The current study aims to examine this speculation, as well as to investigate whether the emotional states of decision-makers and the gain-loss valence of choices moderate the effect of time unpacking on intertemporal decision making. Addressing these issues could contribute to a better understanding of the framing effect in intertemporal decision-making.

### Intertemporal Decision-Making

Intertemporal decision-making has been widely researched in multiple disciplines, such as economics, neuroscience, and psychology. A commonly used research paradigm is to let participants choose between a smaller but sooner (SS) and a larger but later (LL) outcome, for example, between “getting/paying $100 now” and “getting/paying $200 after 6 months.” Using such or similar paradigms, research has frequently shown a temporal preference: people often prefer SS over LL gains and LL over SS losses (Frederick et al., 2002).

Such a temporal preference is often explained by the mental process of temporal discounting (also known as time discounting and delay discounting), which means that the utility or subjective value of an outcome is discounted when delayed (Green and Myerson, 2004). By temporal discounting, a person may perceive that the value of an LL gain (or loss) is smaller than that of an SS gain (or loss), and thus prefer the SS gain (or the LL loss; Green and Myerson, 2004). A series of mathematics models have been proposed to sketch the relationship between the objective and subjective values of future outcomes (e.g., Frederick et al., 2002), such as the widely cited exponential discounting model *V* = *A*e^–δ*D*^ (Samuelson, 1937) and hyperbolic discounting model *V* = *A*/(1 + *kD*) (Ainslie, 1975). In these models, *A* is the amount (i.e., objective value), *V* is the subjective value, *D* is the time delay, and *δ* and *k* are the discount rate of the delayed outcome. According to these models, the subjective value of a given future outcome is determined by the time delay *D* and discount rate *δ* or *k*: A longer time delay and a larger discount rate result in a smaller subjective value (i.e., a larger degree of discounting). Discount rate can be increased by multiple factors, such as longer perceived time intervals, higher perceived time cost, and stronger sensitivity to and psychological impact of the sooner outcomes (Frederick et al., 2002). To clarify, *discount rate* (*δ* or *k*) is different from the term *degree or extent of temporal discounting* used in the current and some other studies (Frederick et al., 2002; Green and Myerson, 2004). By the latter, we refer to the difference in the subjective value of the SS and the LL choices, which is a function of discount rate and time delay.

### The Time Unpacking Effect on Intertemporal Decision-Making

Since intertemporal decision-making involves evaluating and comparing choices at different time points, the perception and estimation of time interval is a critical factor that affects a decision maker’s choices. Studies (Liu and Sun, 2016; Kim and Zauberman, 2019) have found that changing individuals’ time-interval perception can alter their intertemporal choices.

One way to change time-interval perception is to frame it in an unpacking manner, which has been called the time unpacking effect (Kruger and Evans, 2004; Liu and Sun, 2016). To put it in a colloquial language, the unpacking effect means that the whole is less than the sum of its parts (Van Boven and Epley, 2003). It has been broadly investigated under the framework of Support Theory (Tversky and Koehler, 1994). The theory asserts that human judgments of probabilities are attached not to events but to the descriptions of events. The perceived probability of an event (e.g., death from an unnatural death) increases when the event is descriptively unpacked by giving more examples or details (e.g., death from car accidents, homicide, suicide, and fires). Similar effects have been detected for other quantitative judgments, such as the severity of an event’s consequence (Van Boven and Epley, 2003).

The unpacking effect has also been found for time perception (Kruger and Evans, 2004; Liu and Sun, 2016). For example, Kruger and Evans (2004) found that a day was perceived to be longer when specific plans for different timepoints of the day were described than when such details were not described. Liu and Sun (2016) found that a 3-month delay was perceived to be longer when it was described in an unpacking manner (“after the first, second, and then the third month”) than when it was described in a packing manner (“after 3 months”).

The findings of some other studies (Van Boven and Epley, 2003; Tsai and Zhao, 2011) also hint at the existence of the time unpacking effect. In daily life, people often use distinctive, memorable events to mark a point in time or segment a period of time (e.g., “the day when we first met,” “from the day I graduated till the day I get a tenure-track job”). People may feel that time passes faster when they experience such events more intensively during a time period. However, when looking back afterward, they tend to perceive this time period to be longer (Bruss and Rüschendorf, 2010). Even without those landmark events, a time period still could be perceived to be longer when it is divided into a larger number of segments. For example, people tend to feel a 30-month interval becomes longer when it is divided into 10 3-month intervals (Kim and Zauberman, 2019).

To our knowledge, few studies (Liu and Sun, 2016) have examined the time unpacking effect on intertemporal decision making. Liu and Sun (2016) found that participants in the time-unpacking conditions discounted the delayed rewards to larger degrees, thereby showing a stronger preference for immediate over delayed rewards relative to those participants in the time-packing conditions. It is unknown yet whether the time unpacking manipulation interacts with other factors important to intertemporal decision-making, such as the decision-makers’ emotional states and the gain/loss valence of choices.

### The Effect of Emotional State

As reviewed by Herman et al. (2018), a pleasant mood could generally lower discount rate and increase patience (Liu et al., 2013), and an unpleasant mood could increase the discount rate and lead to more impulsive behavior (Augustine and Larsen, 2011; Koff and Lucas, 2011). Emotional state might moderate the effect of time unpacking on intertemporal decision-making through two approaches. On the one hand, emotional state could influence the perception of time interval. Individuals tend to perceive that time is slowing down and time interval is getting longer when they are in an unpleasant (vs. pleasant) state (Guan et al., 2015) and in a high-arousal (vs. low-arousal) state (Droit-Volet et al., 2013). As a result, they are more likely to overestimate the length of time intervals, leading to a stronger preference for SS gains (Kim and Zauberman, 2019).

On the other hand, emotional state could influence the discount rate during decision-making, which is not necessarily associated with time-interval perception. For example, by imagining future emotional events with different valence, empirical studies (Calluso et al., 2019) showed that unpleasant (vs. pleasant) and high-arousal (vs. low-arousal) emotional states tended to make individuals behave more impulsively and discount delayed gains to larger degrees. Moreover, with six experiments, Pyone and Isen (2011) found positive emotions promoted cognitive flexibility, cultivated a higher level of thinking and a more future-oriented view of time, and thereby facilitated participants’ preference for LL gains. Thus, it is reasonable to speculate that emotional state may moderate the time unpacking effect on intertemporal decision-making. The current study manipulated participants’ emotional states by instructing them to imagine future events toned with various emotional valence.

### The Effect of Choices’ Gain-Loss Valence

A myriad of research has demonstrated the essential role of gain-loss valence in decision making (Kahneman and Tversky, 1979). Intertemporal decision-making situations involving losses are as common as those involving gains in daily life, but much less attention has been paid to the former than to the latter in research. The existing studies (e.g., Frederick et al., 2002) have shown that individuals not only discount the subjective value of delayed gains but also that of delayed losses. Namely, a later loss is perceived to be less aversive than a sooner loss of the same amount. The discounting of losses and gains can be fit by similar models (Estle et al., 2006), and the discount rates are usually lower for losses than for gains [known as the sign effect or gain-loss asymmetry (Frederick et al., 2002)]. It may be because losses are more psychologically impactful due to humans’ stronger tendency of loss aversion (Kahneman and Tversky, 1979), and thus are more resistant to mental discounting (Frederick et al., 2002). Thus, choice valence may moderate the effect of time unpacking on intertemporal decision-making by affecting the discount rate. Moreover, choice valence may also affect the perception of time interval (e.g., Bilgin and LeBoeuf, 2010). For instance, with a series of experiments, Bilgin and LeBoeuf (2010) showed that individuals tend to perceive shorter time intervals for delayed losses than for delayed gains.

In summary, the time unpacking manipulation may change individuals’ time-interval perception, thereby altering their perceived value of delayed gains/losses and eventually their choices in intertemporal decision-making tasks. Though yet to be examined, it is possible that the effect of time unpacking on intertemporal choices may be moderated by both decision makers’ emotional states and choices’ gain-loss valence.

### The Current Study

The current study aimed to examine the effect of time unpacking on intertemporal choice, as well as how this effect is moderated by emotional state and choice valence. According to the literature discussed above, we proposed two hypotheses.

H1: Time unpacking manipulation can influence intertemporal choices by prolonging perceived time intervals and thereby downscaling the subjective value of delayed choices (i.e., increasing the degree of temporal discounting).

H2: Emotional state and choice valence can moderate the effect of time unpacking on time perception and the subjective value of delayed choices. An unpleasant mood will reduce the perceived length of time and thus mitigate the time unpacking effect, while a pleasant mood will strengthen this effect.

## Materials and Methods

### Participants

A sample of 87 Chinese college students (54 female; aged between 18 and 27 years, *M* = 21.07, *SD* = 1.59) were recruited to participate in the current study through posters. Three participants were excluded from the analysis due to invalid responses. According to the power analysis by G*Power version 3.1 (Faul et al., 2009), this sample size allowed us to detect an effect *f* = 0.14 (equivalent to *η*^2^*_p_* = 0.020) in our design with a power of 1−β = 0.80 at a level of *α* = 0.05, which was between a small (*f* = 0.10) and a medium (*f* = 0.25) effect sizes (Cohen, 1977). Participants gave informed consent before the experiment and were debriefed with the research purpose after the experiment. By completing the experiment, each participant received RMB 10 yuan (around 1.44 US dollars) as compensation. The study received ethical approval from the Ethics Committee of the Faculty of Education at Hubei University.

### Tasks and Measurement

#### Induction and Measurement of Emotion

Emotional states were induced by an episodic-thinking (ET) task. Research has found that imagining pleasant events (the pleasant ET condition) can evoke pleasant emotions, while imagining unpleasant events (the unpleasant ET condition) can trigger unpleasant emotions (Wang et al., 2012). Specifically, we presented each participant with one of three event lists: pleasant-event list (winning a scholarship, holding a wedding, receiving a gift, attending a wedding with good friends, and passing an exam), neutral-event list (washing clothes, washing hair, brushing teeth, washing feet, and washing face), and unpleasant-event list (fighting with a good friend, food poisoning, attending a relative’s funeral, arguing with parents, and a car accident). Participants were free to choose one of the five events on the list and then imagined themselves experiencing this event at a moment in the future. It has been shown that these events are often perceived as common and personally relevant by Chinese young adults (Liu et al., 2013). After the ET task, participants self-reported whether they had imagined the event as instructed (1 = *no*; 2 = *yes*); they also rated the vividness of imagination (from 1 = *not vivid at all* to 7 = *very vivid*), emotional valence of the event (from 1 = *very unpleasant* to 7 = *very pleasant*), emotional arousal of the event (from 1 = *very low* to 7 = very high), and personal relevance of the event (from 1 = *totally irrelevant* to 7 = *totally relevant*).

The Chinese version (Qiu et al., 2008) of the Positive and Negative Affect Schedule (PANAS) was used to measure participants’ emotions before the ET (i.e., pretest/baseline emotions) and at the end of the whole experiment (i.e., posttest emotion). The scale consisted of nine words describing pleasant emotions (i.e., active, energetic, happy, elated, excited, proud, joyful, vigorous, and grateful) and nine words for unpleasant emotions (i.e., shameful, sad, scared, nervous, terrified, guilty, irritable, trembled, and angry). For each emotion, participants rated the extent to which they were experiencing it at that moment on a five-point Likert scale (from 1 = *not at all* to 5 = *extremely*). Cronbach’s α of the pre- and post-test was 0.90 and 0.96 for the positive affect subscale, and 0.92 and 0.95 for the negative affect subscale.

#### Manipulation and Measurement of Time Perception

Following the previous studies (Liu and Sun, 2016), time perception for the delay was altered through time-unpacking manipulation. In the time-unpacking condition, the LL option in the intertemporal choice tasks was “From now on, after passing through the 1st, 2nd, 3rd, 4th, 5th, and 6th month, get (or pay) 1,000 yuan.” In the time-packing condition, the LL option was “Get (or pay) 1,000 yuan after 6 months.” After the choice tasks, participants reported their perceived time delay (i.e., subjective delay) of the LL option by rating on a continuous scale ranging between 1 and 100 (Liu and Sun, 2016).

#### Intertemporal Choice Task

Each participant completed four blocks of intertemporal choice-making task, each consisting of 19 trials. The four blocks corresponded to the four “time unpacking × choice valence” conditions (i.e., the time-packing gain condition, time-packing loss condition, time-unpacking gain condition, and time-unpacking loss condition). Similar to previous studies (Rachlin et al., 1991), in each trial participants chose between two options: An SS option “Get (or pay) *x* yuan now” and an LL option “Get (or pay) 1,000 yuan after 6 months” (time-packing) or “From now on, after passing through the 1st, 2nd, 3rd, 4th, 5th, and 6th month, get (or pay) 1,000 yuan” (time-unpacking). The amount of gain (or loss) in the LL option remained the same, while that in the SS option (i.e., *x*) increased from 50 yuan to 950 yuan in order with a step of 50 across the 19 trials within a block.

### Experimental Design and Procedures

The experiment adopted a mixed 3 (ET: pleasant, neutral, or unpleasant; between-subject) × 2 (time unpacking: yes or no; within-subject) × 2 (choice valence: gain or loss; within-subject) design. Participants were randomly assigned to the pleasant (*n* = 31), unpleasant (*n* = 30), and neutral ET conditions (*n* = 26) to complete the experiment run by E-prime 2.0 on computer.

**Figure 1:**
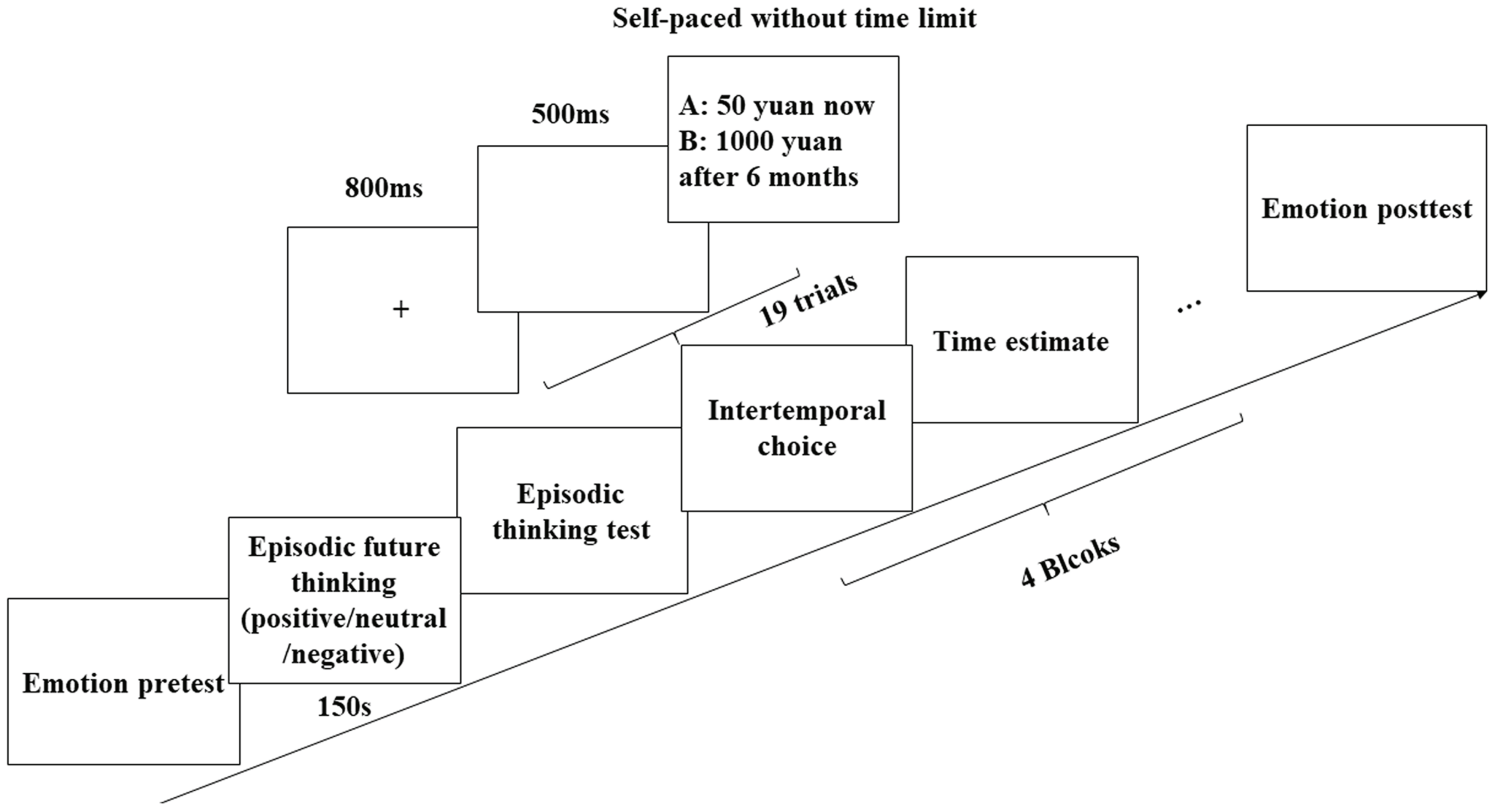
A flow diagram showing the procedures of the experiment.

As displayed in Figure 1, participants first completed the Chinese version of the PANAS (Qiu et al., 2008), which measured their current emotional state (pretest emotions). Afterward, they were instructed to choose an event from one of the event lists (the pleasant, neutral, or unpleasant event list) and imagine it vividly for 2 min (i.e., the ET task), and then they answered several questions checking their engagement in the ET task.

Next, participants completed four blocks of intertemporal choice task, each corresponding to a time packing × choice valence condition (i.e., the time-packing gain condition, time-packing loss condition, time-unpacking gain condition, or time-unpacking loss condition). A fixed sequence among the blocks was adopted, that is, time-packing gain, time-packing loss, time-unpacking gain, and time-unpacking loss. Each block consisted of 19 trials and all trials used a fixed sequence from small to large. In each trial, participants watched a screen with a cross at its center lasting for 800 ms, followed by a blank screen lasting for 500 ms, and then an intertemporal choice task. Participants were instructed to make a choice by pressing certain keys on the keyboard without a time limit. After finishing all 19 trials of choice tasks in each block, participants rated their perceived time delay for the LL option (which was the same for all trials within a block) in the block.

At the end of the experiment, participants completed the Chinese version of the PANAS again that measured their current emotional states (posttest emotions).

### Analytic Strategies

For each experimental condition of each participant, the subjective value of the LL option equaled to the mean value of the last LL option and the first SS option when a participant shifted his or her choice from the LL to SS options in the ordered sequence of intertemporal choice-making trials. For example, if the participant choose the LL option for the fourth trial (SS = 200 yuan, LL = 1,000 yuan) but the SS option for the fifth trial (SS = 250 yuan, LL = 1,000 yuan) in a task block, then the subjective value of the LL option (i.e., the delayed 1,000 yuan) was (200 + 250)/2 = 225 yuan.

To test our hypotheses, the subjective time delays and subjective values of the LL options were submitted to the *Afex* (Singmann et al., 2020) and *emmeans* (Lenth, 2021) packages on R version 3.6.0 (R Core Team, 2020) for a mixed design repeated measures 3 (ET: pleasant, neutral, or unpleasant; between-subject) × 2 (time unpacking: yes vs. no; within-subject) × 2 (choice valence: gain vs. loss; within-subject) ANOVA. In addition, the validity of the emotion manipulation was also checked.

## Results

### Demographics

The demographic information of the three ET conditions is displayed in Table 1. ANOVAs showed no significant differences in age, *F*(2, 84) = 1.25, *p* = 0.29, *η*^2^*_p_* = 0.03, years of schooling, *F*(2, 84) = 1.10, *p* = 0.34, *η*^2^*_p_* = 0.03, monthly consumption level, *F*(2, 84) = 0.60, *p* = 0.55, *η*^2^*_p_* = 0.01, or urgency of needing money, *F*(2, 84) = 0.89, *p* = 0.41, *η*^2^*_p_* = 0.02, across the three conditions. Chi-square tests showed no significant differences among the three conditions in gender ratio, *χ*^2^(2) = 1.60, *p* = 0.40, or major of study, *χ*^2^(6) = 5.2, *p* = 0.50.

**Table 1:** Sample characteristics.

Demographic variables	Pleasant condition	Neutral condition	Unpleasant condition
Gender (m:f)	11:20	8:18	14:16
Age (year)	21.29 ± 1.64	21.23 ± 1.73	20.7 ± 1.39
Education (year)	15.23 ± 0.88	15.42 ± 1.3	15 ± 1.02
**Major**			
Engineering	2	5	4
Science	13	13	14
Literature	14	7	12
Art	2	1	0
Monthly consumption (RMB yuan)	1454.84 ± 494.53	1653.85 ± 936.90	1636.67 ± 862.83
Urgency of needing money	2.65 ± 1.40	2.85 ± 1.29	3.10 ± 1.30

### Check of Emotion Manipulation

All participants reported that they had imagined the event in the ET task as instructed. Participants’ ratings on the features of the imagined events are displayed in Table 2. ANOVAs showed no significant differences in vividness, *F*(2, 84) = 1.18, *p* = 0.31, *η*^2^*_p_* = 0.03, or personal relevance, *F*(2, 86) = 1.02, *p* = 0.37, *η*^2^*_p_* = 0.02, but significant differences in emotional valence, *F*(2, 86) = 134.33, *p* < 0.001, *η*^2^*_p_* = 0.76, and arousal of the event, *F*(2, 86) = 15.24, *p* < 0.001, *η*^2^*_p_* = 0.27. *Post hoc* tests revealed that emotional valence was the highest in the pleasant and lowest in the unpleasant ET condition (*p*s < 0.001). Arousal was higher in the pleasant and unpleasant conditions than in the neutral condition (*p*s < 0.001). The results indicated that participants were engaged in the ET task as anticipated.

**Table 2:** Results of episodic thinking (ET) in different conditions (*M* ± *SD*).

Features	Pleasant condition	Neutral condition	Unpleasant condition
Vividness	5.16 ± 1.27	4.81 ± 1.27	4.67 ± 1.35
Event valence	5.74 ± 0.96	4.38 ± 0.75	2.23 ± 0.77
Event arousal	5.16 ± 1.04	3.73 ± 0.96	4.83 ± 1.02
Relevance	6.23 ± 0.96	5.88 ± 0.95	6.07 ± 0.78
Pretest PA	28.90 ± 4.35	25.12 ± 6.40	28.06 ± 6.02
Pretest NA	17.32 ± 6.58	17.77 ± 6.07	17.03 ± 7.07
Posttest PA	26.74 ± 7.42	22.65 ± 7.28	21.30 ± 5.57
Posttest NA	13.19 ± 4.66	14.00 ± 5.86	16.20 ± 6.74

PA, positive affect; NA, negative affect.

To test if the ET task induced specific emotions in participants, repeated measures 3 (ET: pleasant, neutral, or unpleasant; between-subject) × 2 (timepoint: pretest vs. posttest; within-subject) ANOVAs on participants’ pleasant and unpleasant emotions (measured by the Chinese version of the PANAS) were performed. For pleasant emotions, there was significant main effects of timepoint, *F*(1, 84) = 29.68, *p* < 0.001, *η*^2^*_p_* = 0.26, and ET condition, *F*(2, 84) = 4.50, *p* = 0.014, *η*^2^*_p_* = 0.10, as well as a significant ET × timepoint interaction, *F*(2, 84) = 4.69, *p* = 0.012, *η*^2^*_p_* = 0.10. Simple effect analysis revealed that, at the posttest than at the pretest, participants’ pleasant emotions were slightly higher in the pleasant ET condition, *F*(1, 84) = 3.45, *p* = 0.067, *η*^2^*_p_* = 0.04, slightly higher in the neutral ET condition, *F*(1, 84) = 3.75, *p* = 0.056, *η*^2^*_p_* = 0.04, and lower in the unpleasant ET condition, *F*(1, 84) = 32.71, *p* < 0.001, *η*^2^*_p_* = 0.28. For unpleasant emotions, the results showed a significant main effect of timepoint (i.e., less unpleasant at the posttest than the pretest), *F*(1, 84) = 16.651, *p* < 0.001, *η*^2^*_p_* = 0.165, while the main effect of the ET condition, *F*(2, 84) = 0.509, *p* = 0.603, *η*^2^*_p_* = 0.012, and the ET × timepoint interaction, *F*(2, 84) = 2.211, *p* = 0.116, *η*^2^*_p_* = 0.050, were non-significant.

Contradicting our expectation, after the ET task, the pleasant emotions did not increase in the pleasant-ET condition (although they decreased in the unpleasant-ET condition), and the unpleasant emotions decreased in all three ET conditions. These results show that imagining future events did not effectively induce emotions. However, we still included the variable ET (pleasant, neutral, or unpleasant) in the follow-up analyses to control for the potential impacts of emotion manipulation on the outcome variables of interest.

### Effects on Time Perception

The 3 (ET: pleasant, neutral, or unpleasant; between-subject) × 2 (time unpacking: yes vs. no; within-subject) × 2 (choice valence: gain vs. loss; within-subject) ANOVA on subjective time showed that only the main effect of time unpacking was significant, *F*(1, 81) = 7.76, *p* = 0.007, *η*^2^*_p_* = 0.09, 95% CI of *η*^2^*_p_* = (0.014, 0.193). The subjective time in the time packing condition (*M* = 47.8) was significantly shorter than that in the time unpacking condition (*M* = 52.5), indicating that the time-unpacking operation effectively lengthened the subjective time. Choice valence and emotional manipulation did not moderate the effect of time unpacking on time perception, as indicated by the non-significance of interactions between time unpacking and choice valence and between time unpacking and ET (*ps* > 0.10). The results were consistent with our hypothesis H1.

### Effects on the Subjective Values of Delayed Choices

**Figure 2:**
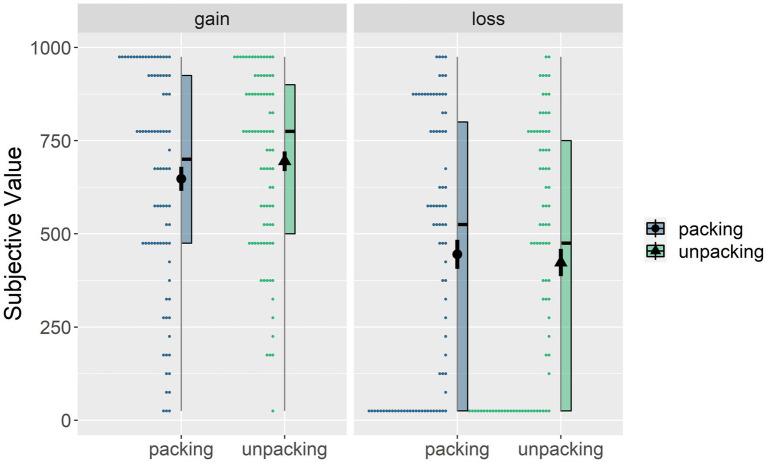
The interaction effect of time unpacking (packing vs. unpacking) and choice valence (gain vs. loss) on the subjective values of delayed choices. The points in the figure represent the results of each trial. The line is the range of dependent variables. The colored rectangles represent the 25, 50, and 75% quartiles, respectively, and the black dots and line segments represent the mean and SE.

The 3 (ET: pleasant, neutral, or unpleasant; between-subject) × 2 (time unpacking: yes vs. no; within-subject) × 2 (choice valence: gain vs. loss; within-subject) ANOVA on the subjective value showed a significant main effect of choice valence (the gain conditions > the loss conditions), *F*(1, 81) = 31.37, *p* < 0.001, *η*^2^*_p_* = 0.279, 95% *η*^2^*_p_* CI = (0.147, 0.396) and a non-significant main effect of time unpacking manipulation, *F*(1, 81) = 0.42, *p* = 0.52, *η*^2^*_p_* = 0.005, 95% CI of *η*^2^*_p_* = (0, 0.059). The interaction effect of time unpacking × choice valence was significant, *F*(1, 81) = 4.432, *p* = 0.038, *η*^2^*_p_* = 0.052, 95% CI of *η*^2^*_p_* = (0.001, 0.146). As shown in Figure 2, simple effects tests for the time unpacking × choice valence interaction revealed that the effect of time unpacking was significant only for gains, *F*(1, 81) = 5.133, *p* < 0.026, *η*^2^*_p_* = 0.06, 95% CI of *η*^2^*_p_* = (0.004, 0.157), partially supporting our hypothesis H2 (Table 3).

**Table 3:** Subjective values of delayed choices in different conditions (*M* ± *SD*).

	Packing	Unpacking
Gain	648 ± 295	695 ± 241
Loss	445 ± 358	423 ± 337

## Discussion

With an experimental design, the current study found that time unpacking manipulation lengthened the perceived time delay during intertemporal decision-making, regardless of the manipulation of decision-makers’ emotional states, and the gain-loss valence of choices. The time unpacking manipulation also impacted the valuation of the delayed choices: participants discounted the delayed gains (but not losses) less (and thus perceived more values from the delayed gains) when the delay was unpacked than when the delay was not unpacked. The results support H1 and partially support H2.

Replicating the time unpacking effect (e.g., Kruger and Evans, 2004; Liu and Sun, 2016), the current study showed that describing a time interval as a sequence of smaller steps prolonged the length of the interval participants perceived. This effect was robust and not moderated by choice valence, which has been frequently found to impact time perception (e.g., Bilgin and LeBoeuf, 2010; Droit-Volet et al., 2013). Tversky and Koehler (1994) suggested that the time unpacking effect is due to attentional bias. Tse et al. (2004) examined the relationship between attention and temporal distance perception of novel stimuli. They found that subjects with both visual and auditory stimuli tended to judge the presentation time of novel stimuli as being longer than that of standard stimuli. Similarly, temporal decomposition descriptions are uncommon to subjects and therefore its occurrence can overestimate temporal distance (Liu and Sun, 2016).

Contrary to our expectation and previous studies (e.g., Liu and Sun, 2016), time unpacking manipulation did not decrease, but increase the subjective value of delayed gains. In other words, time unpacking manipulation increased participants’ perceived time length but decreased their degrees of temporal discounting. Typically, the degree of temporal discounting for a delayed outcome should increase when the perceived time delay prolongs (Kim and Zauberman, 2019). These results suggest that time unpacking manipulation not only impacts time perception but also some other factors, which, in turn, influence the valuation of delayed outcomes and thereby intertemporal choices. Future studies are needed to further explore what these factors could be.

The sense of control over the delayed outcome could be one of these other factors mentioned above. Support theory claims that the probability of a multifaceted category increases and becomes more supportive when the category is unpacked into its components (Tversky and Koehler, 1994). When a period of time is decomposed into several shorter periods, the degree of belief in the longer time perception of this period will be increased. According to the construal level theory (Trope and Liberman, 2003), explicit components are more convincing and can dominate distant behavioral though they have lower construal level (Eyal et al., 2004; Herzog et al., 2007). Therefore, a delayed gain full of explicit components under a time unpacking condition reminds people of possibilities that would not have been considered otherwise, resulting in a lower discount rate. This could be the reason why participants discounted the delayed gains to lower degrees in the unpacking than the packing condition even though the time unpacking condition increased participants’ perceived length of delay.

With regard to losses, the time unpacking effect on intertemporal choice was not found. It could be that losses tend to be more psychologically impactful than gains (e.g., Kahneman and Tversky, 1979). According to the endowment effect (Thaler, 1980), people endow the properties they own with high value and are highly averse to losses of the properties. People’s strong loss aversion may make them focus more on the value of losses and overlook other information such as time. In line with this speculation, previous studies (e.g., Frederick et al., 2002) have shown that the delay discounting effect is usually smaller for losses than for gains.

Out of our expectation, the emotion-manipulation task (i.e., asking participants to imagine future events) did not effectively induce specific emotional states, although it has been frequently adopted in previous studies (Wang et al., 2014; Calluso et al., 2019; Wang and He, 2019). One of the possible reasons was that the interval between the pretest and posttest of emotional states might have been too long for the manipulation effects to be sustained throughout our study.

## Limitations and Conclusion

One limitation of the current study was that we only included a single time delay (i.e., a total of 6 months for both the time packing and unpacking conditions) and a fixed magnitude (RMB 1,000 yuan) for the delayed choices. Both the magnitudes and time delays of choices could have modulated temporal discounting (e.g., Frederick et al., 2002). Future studies need to take these factors into account to give a full picture of the time unpacking effect and its interaction with emotion and choice valence. The second limitation was that in this study, titration was used to measure subjective value, and titration was measured in an increasing order, which had an order effect. Moreover, the order between groups was fixed which may have produced a sequence effect. The third limitation was that emotional states were not successfully manipulated. More effective methods should be adopted to manipulate emotional states in future studies.

Despite these limitations, the current study provides supportive evidence for the relatively newly identified time unpacking effect. Namely, time unpacking prolongs the perceived length of time durations. Contradicting some previous studies, the current study showed smaller degrees of temporal discounting in the time-unpacking condition as compared to the time-packing condition, suggesting that time unpacking may influence temporal discounting by impacting multiple factors (but not only time perception). The current study also showed that the effect of time unpacking on temporal discounting could be moderated by contextual factors such as the gain-loss valence of choices. Taken together, these results highlight the complexity of the time unpacking effect on intertemporal decision-making and the need for more research on such an effect.

## Data Availability Statement

The datasets presented in this study can be found in online repositories. The names of the repository/repositories and accession number(s) can be found at: https://osf.io/48bx5/.

## Ethics Statement

The studies involving human participants were reviewed and approved by the Ethics Committee of the Faculty of Education in Hubei University. The patients/participants provided their written informed consent to participate in this study. Informed consent was obtained from all individual adult participants included in the study.

## Author Contributions

QY analyzed the data and wrote the draft. XG and JX participated in paper writing. SY developed research question and designed the study. All authors contributed to the article and approved the submitted version.

### Conflict of Interest

The authors declare that the research was conducted in the absence of any commercial or financial relationships that could be construed as a potential conflict of interest.
